# Supplementary material for: Robust adaptive optics for localization microscopy deep in complex tissue
Source: Nat Commun. 2021 Jun 7;12:3407. doi: 10.1038/s41467-021-23647-2 (PMC8184833; doi:10.1038/s41467-021-23647-2)
Supplement: Supplementary file 2 — Supplementary information [file 41467_2021_23647_MOESM2_ESM.pdf]

Supplementary Information for:

Robust adaptive optics for localization microscopy deep in complex tissue

Marijn E. Siemons[1], Naomi A.K. Hanemaaijer[1,2], Maarten H.P. Kole[1,2], Lukas C. Kapitein[1]

Supplementary Figure 1

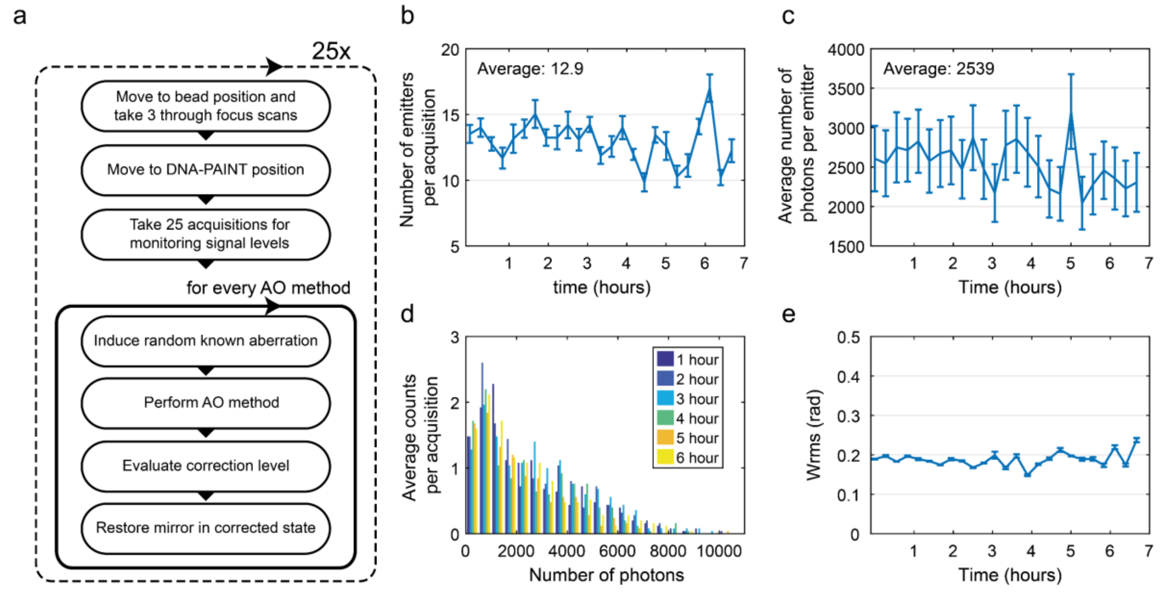

DNA-PAINT sample and deformable mirror remain stable during the experiment of figure 1f. a) Schematic of the experimental procedure, consisting of a monitoring part (first 3 blocks) and a correction part (bottom 4 blocks). For all AO methods tested, the stage moves to a position with a bead and acquires 3 through-focus scan. Next, 25 frames were acquired at the DNA-PAINT position (without a bead in the FOV) with the system-corrected DM state. From these acquisitions the number of emitters (b), number of photons per emitter (c) and distribution of photon counts were measured (d). This revealed that the signal levels remained constant for the full 7 hour duration of the experiment. The acquired through-focus scans (in system corrected state) are analyzed with a phase retrieval algorithm [25] to check for possible drift in the mirror. The aberration level (e) remained at a level of 0.2 rad RMS during the whole experiment. Error bars indicate the standard error of mean in the number of emitters (b), number of photons (c) of the 25 frames and estimated aberration level (e) measured from 3 through focus scans.

Supplementary Figure 2

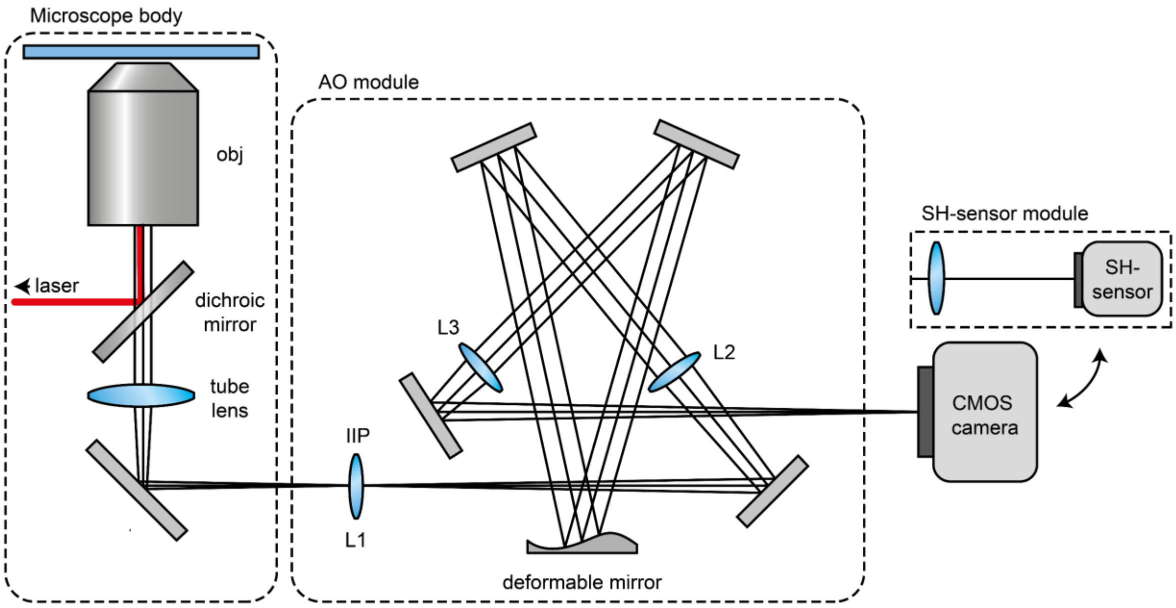

Illustration of the set up. The AO module consists of a 4F system (lenses L2 and L3, *f* = 500 mm), where the deformable mirror (DM) is placed in the back focal plane of L2. Another lens (L1, *f* = 750 mm) is placed in the intermediate image plane (IIP), which conjugates the pupil plane of the objective to the DM. This lens is needed as the tube lens and objective inside the microscope body are placed approximately 5 cm too close to each other to form a 4F system. For calibration of the DM, the CMOS camera is replaced with the SH-sensor module, which consists of a lens (*f* = 100 mm) and a Shack-Hartman sensor.

Supplementary Figure 3

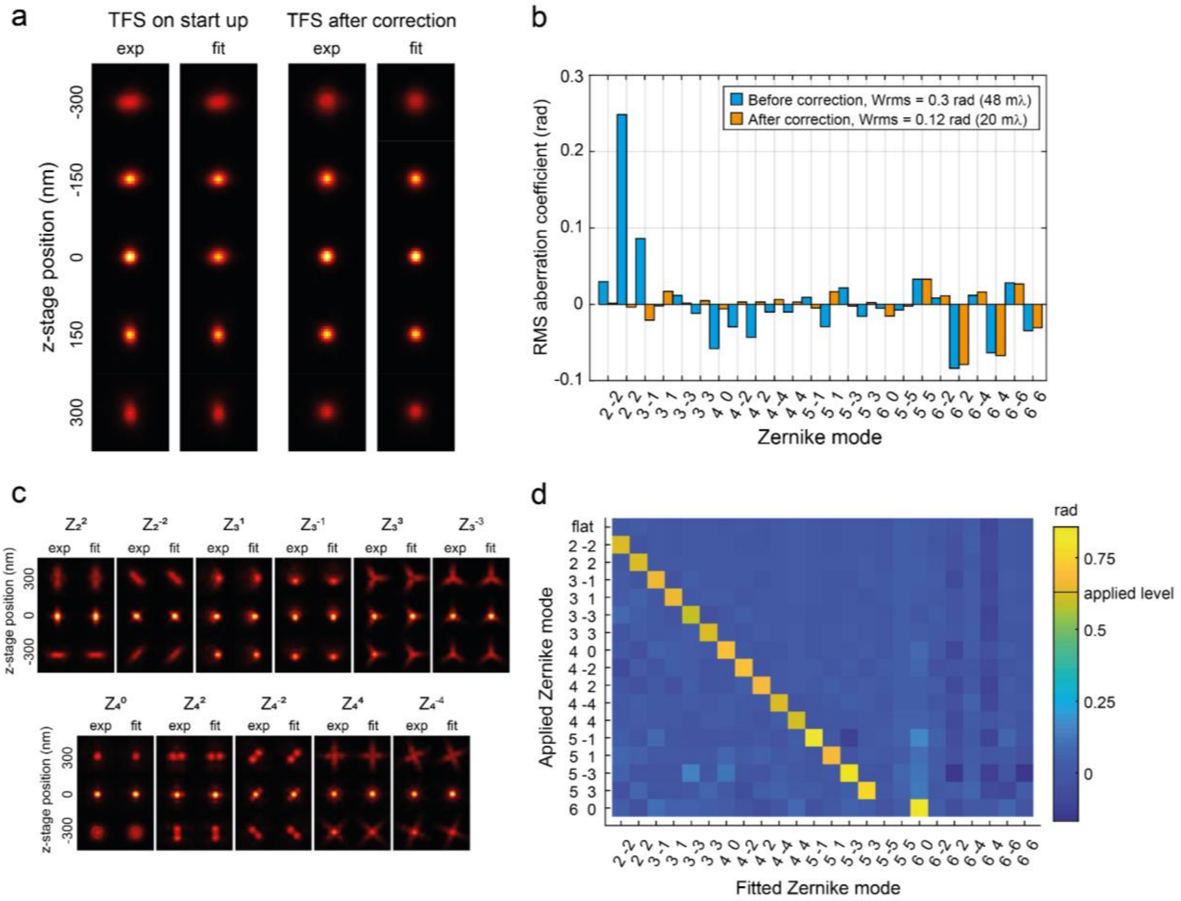

Verification of the DM-calibration. a) On start-up the experimental PSF (exp) is aberrated due to drift in the deformable mirror. A phase retrieval algorithm (fit) (see methods) estimated the specific Zernike modes as shown in (b). The inverse Zernike coefficients of modes Z2±2, Z3±1, Z3±3, Z40, Z4±2, Z4±4, Z5±1, Z5±3 and Z60 were subsequently applied by the mirror, which improves the PSF (TFS after correction). Phase retrieval revealed that all major contributing Zernike modes were nullified. c) PSFs and phase retrieval fits corresponding to Zernike modes Z2±2, Z3±1, Z3±3, Z40, Z4±2, Z4±4 with an amplitude of 0.63 rad (100 mλ). d) phase retrieval of (c) revealed that this DM is capable of accurately modulating Zernike modes up to the fourth order with little crosstalk. Higher order modes such as Z5±1, Z5±3 and Z60 appear to have crosstalk with lower modes.

Supplementary Figure 4

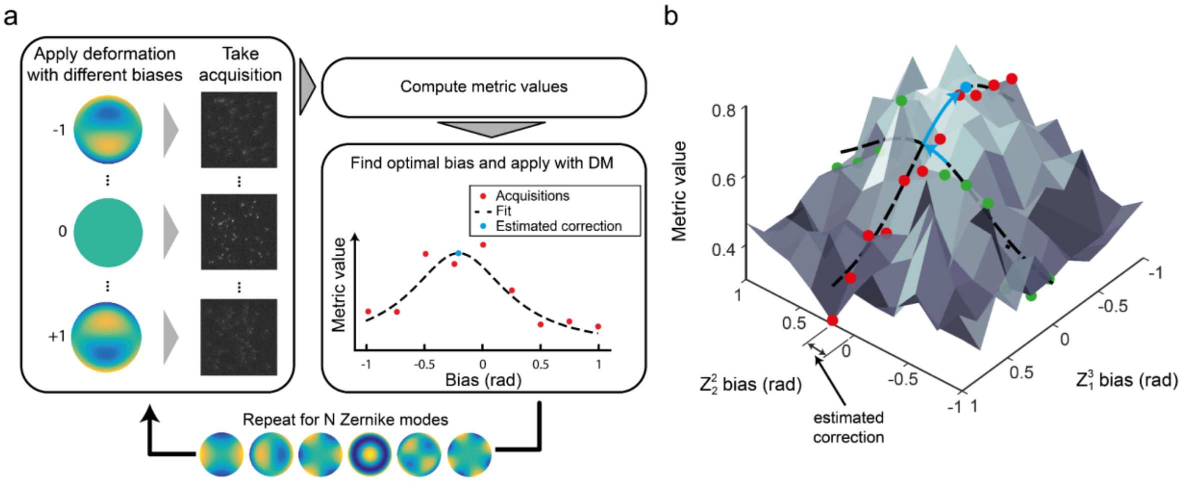

Schematic of model-based optimization. **a**) For each Zernike mode a series of frames is acquired with different biases (amplitude of the Zernike mode). From these acquisitions the metric value is computed. Next, the optimal bias is determined by fitting an appropriate function (the metric curve) to these points, after which the estimated correction is applied. This is performed for multiple Zernike modes. **b**) The optimum correction for Zernike modes can be more easily estimated when the contrast in metric value is improved. Therefore it is beneficial to first correct major expected types of aberrations, such as spherical aberration. For the same reason, multiple correction rounds also yield additional improvement.

Supplementary Figure 5

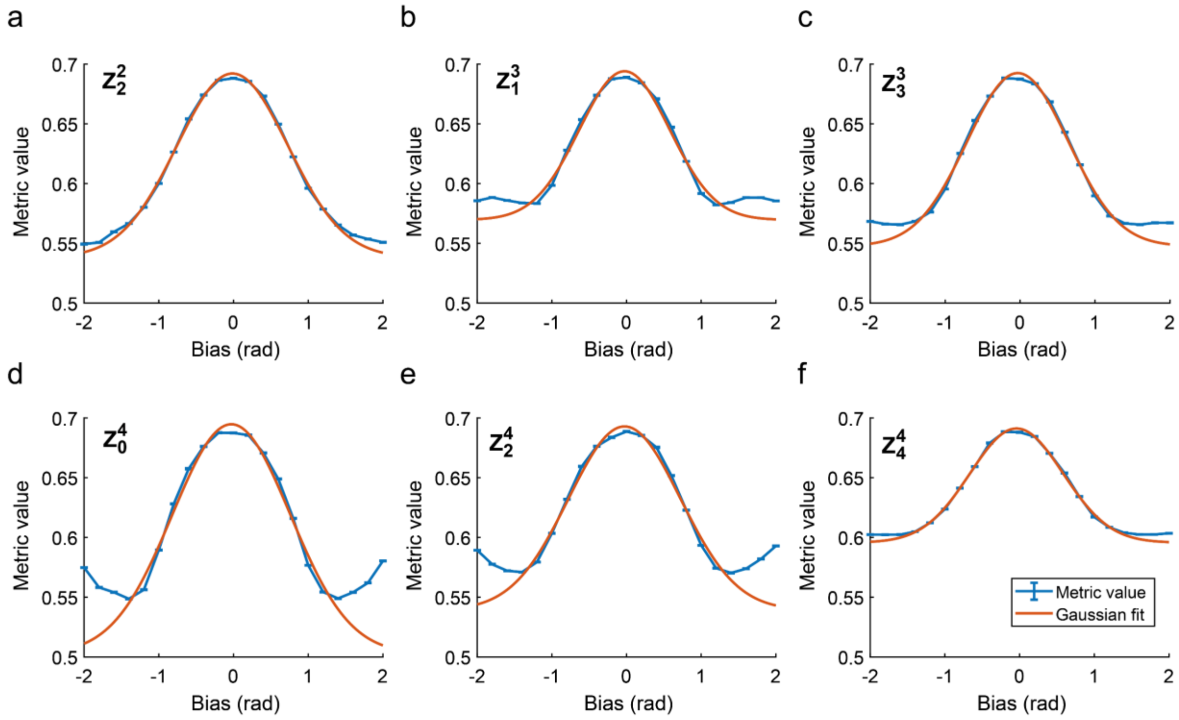

Metric curves for different Zernike modes. a-f) Metric value as function of applied bias for different Zernike modes. These values were obtained by simulating single-molecule acquisitions (see methods) with different biases for each Zernike mode. A Gaussian function with offset properly describes the metric values to all Zernike modes up to the 4th order in a ±1 rad range and was therefore used as the metric curve. Increasing the bias beyond ±1.5 rad results in an increase in the metric value for some Zernike modes. This inversion of the metric value occurs due to contrast inversion at (specific) spatial frequencies.

Supplementary Figure 6

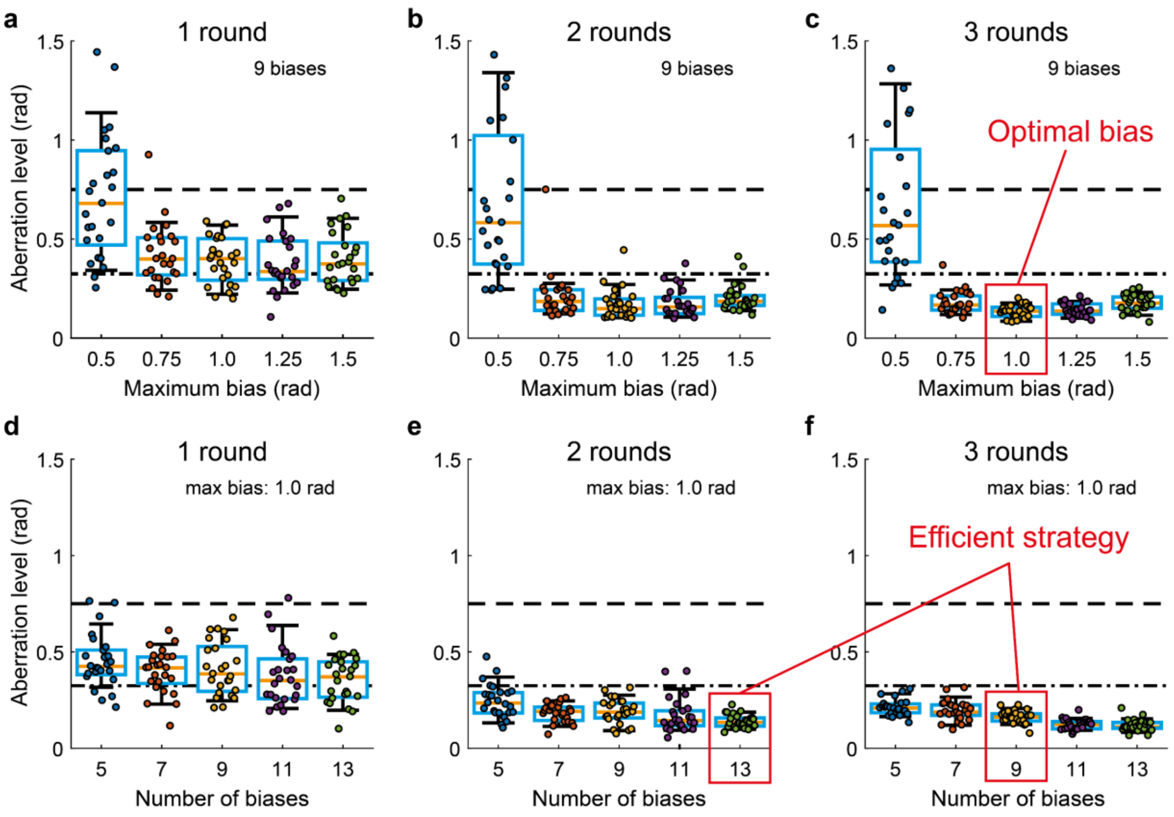

Simulation-based optimization of REALM. a-c) residual aberration level for different maximum applied biases and correction rounds, using 9 biases per Zernike mode (11 Zernike modes in total). Dashed line indicates induced aberration level (0.75 rad RMS). Residual aberrations are minimal when using a bias of 1 rad. d-f) Residual aberration level for different number of biases and correction rounds. Approaches with 13 biases in 2 correction rounds (total of 286 acquisitions for 11 Zernike modes) or 9 biases in 3 correction rounds (total 297 acquisitions for 11 Zernike modes) both constitute efficient strategies to achieve robust correction. All simulations contain 25 random aberration configurations (n = 25) for each optimization configuration. Box plot indicates 9/91-percentile, 25/75-percentile and median.

Supplementary Figure 7

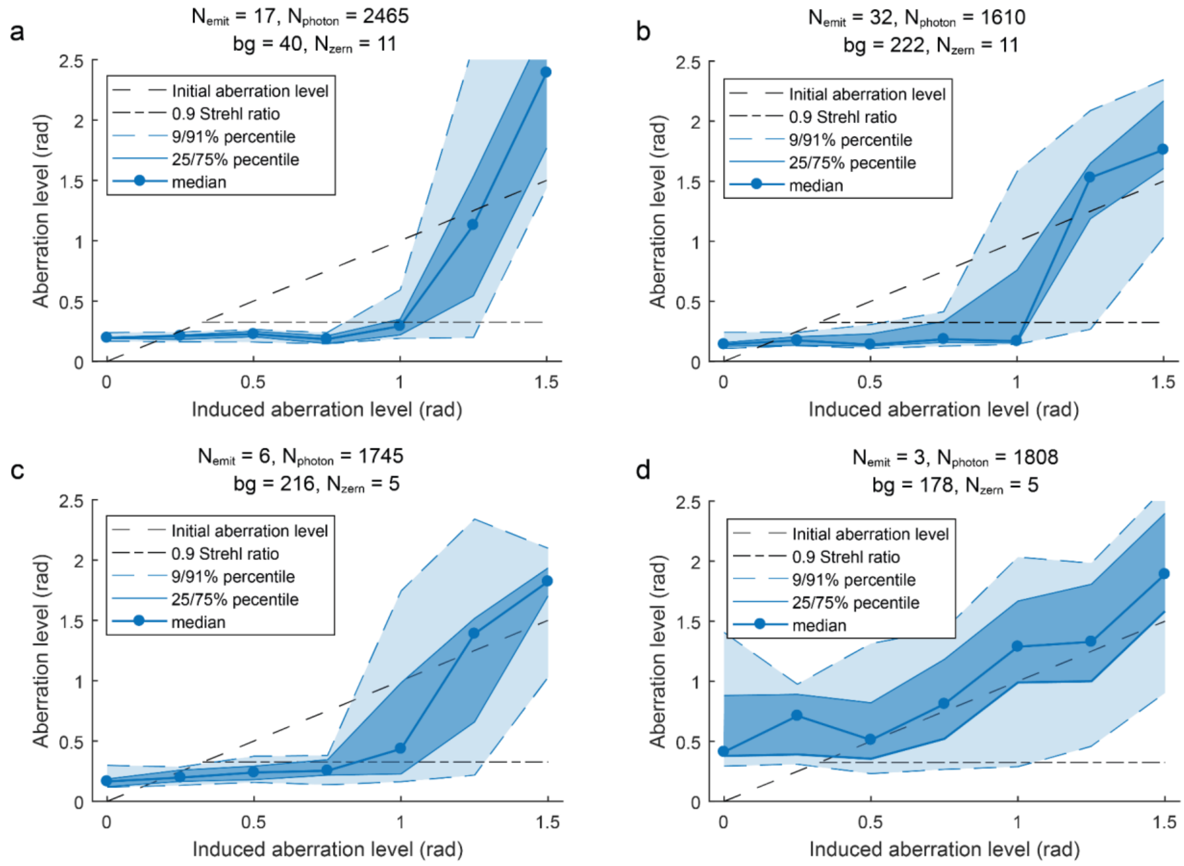

a-d) Experimental performance of REALM as function of induced aberration level using a DNA-PAINT sample in different signal to background ratio's (SBR). Here 2 correction rounds with 13 biases per Zernike mode with a maximum bias of 1 rad is used. For each aberration level, 10 random aberration configurations were induced by the deformable mirror and subsequently corrected. Results below the initial aberration level (dashed line) indicate improvement, results below 0.9 Strehl-ratio (dashed-dotted line) indicate proper imaging conditions. For (a) and (b) these aberration configurations consisted of 11 Zernike modes (astigmatism, coma, primary spherical aberration, trefoil, second order astigmatism and quadrafoil) and for (c) and (d) these consisted of 5 Zernike modes (astigmatism, coma and primary spherical aberration). The measured total signal to background ratio (SBR) was 0.0065 (a), 0.0015 (b), 0.0003 (c) and 0.0002 (d). The signal and background level are estimated from 25 pre-acquisitions before an aberration is induced. N_{emit}, N_{photon}, bg and N_{zern} indicate the average number of emitters per frame, the average number of emitted photons of each emitter, the background photon count and the number of applied Zernike modes present in the aberration configuration. Error bands indicate 9/91% and 25/75% percentile in all panels.

REALM was capable of correcting up to 1 rad RMS of wave-front error when the aberration was completely random for all but the lowest SBR. In practice, a major contribution is due to spherical aberration, which can be roughly pre-corrected, significantly increases the aberration level which can be corrected by REALM.

Supplementary Figure 8

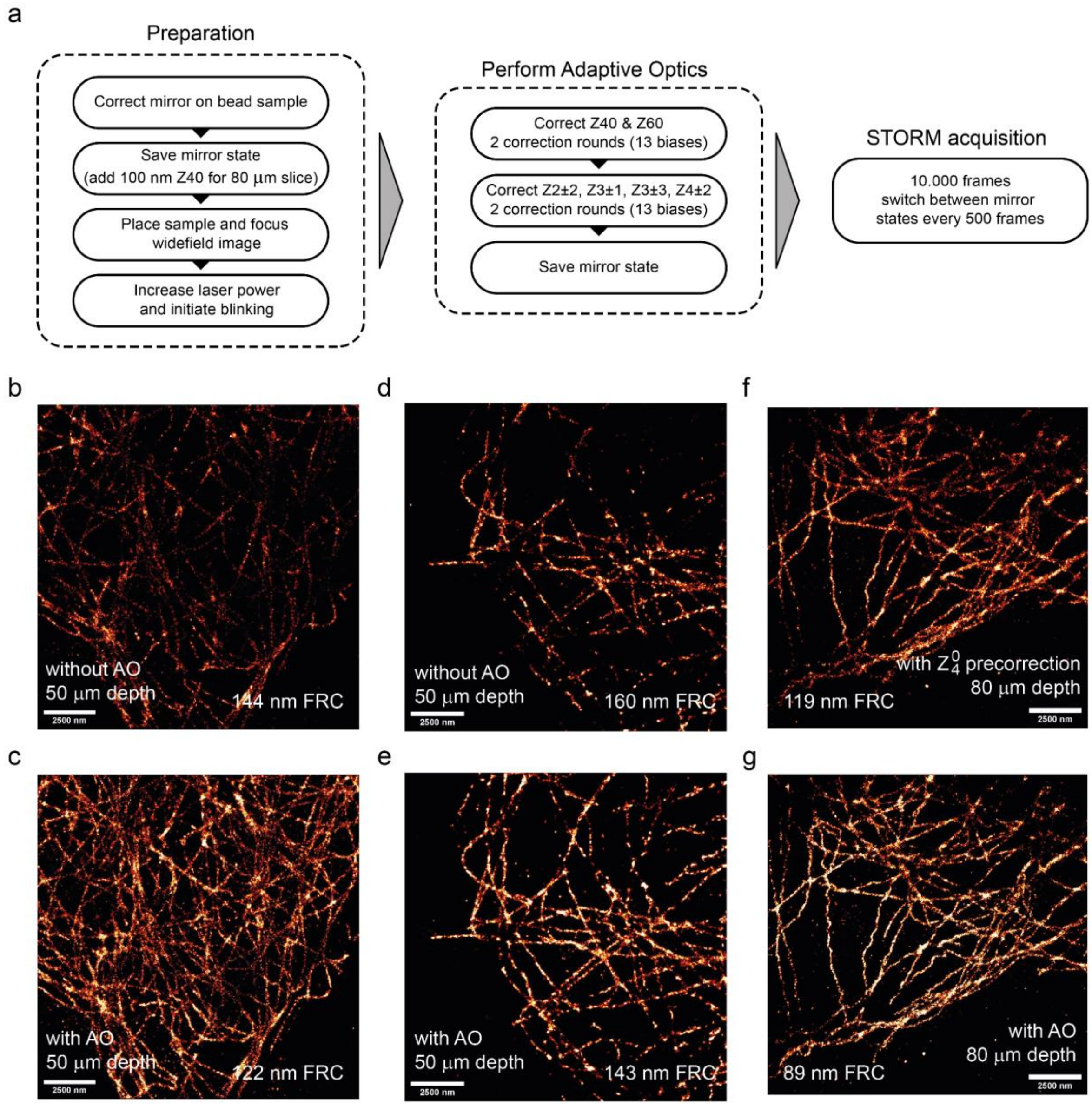

a) Experimental procedure for comparing SMLM with and without adaptive optics. First, the mirror was used to correct all system induced aberrations using a bead sample (see Supplementary Figure 2). This mirror state was saved and later used as the system-corrected DM state. For the 80 µm slice we pre-corrected spherical aberration by applying this mode with an amplitude of 100 nm (0.9 rad) (RMS normalized amplitude). Next, the sample was mounted and focused using widefield imaging, while trying to keep the illumination minimal. Subsequently, the laser power was increased to initiate the blinking and spherical aberration was corrected, followed by the other Zernike modes. After correction the mirror state was saved as sample-corrected state and the SMLM acquisition was started. During this acquisition the mirror switches between the system- or *Z*40 pre-corrected and sample-corrected state every

500 frames. b) SMLM reconstruction of microtubules in COS-7 cells imaged through a 50 μm thick brain section using the frames with the DM in system-corrected state (without AO). Image contains a total of 34K successful localizations with a localization precision <20 nm. c) SMLM reconstruction of b) using frames with sample-corrected DM state. The estimated aberration level was 0.81 ± 0.02 rad RMS and 123K events were successfully localized with a precision <20 nm. d&e) Another example as b&c, consisting of 17K localizations (without AO) and 53K localizations (with AO) with a localization precision <20 nm. Estimated aberration level was 1.07±0.08 rad RMS. f) SMLM reconstruction of microtubules in COS-7 cells imaged through a 80 μm thick brain section using the frames with a precorrection of spherical aberration (35K localizations with a localization precision <20 nm). g) as (f) but with frames with the DM in sample corrected state (79K localizations with a localization precision <20 nm). Estimated aberration level was 0.91 ± 0.04 rad RMS (on top of 0.9 rad pre-corrected spherical aberration). Repeated n=10 times in 3 distinct samples with similar results.

Supplementary Figure 9

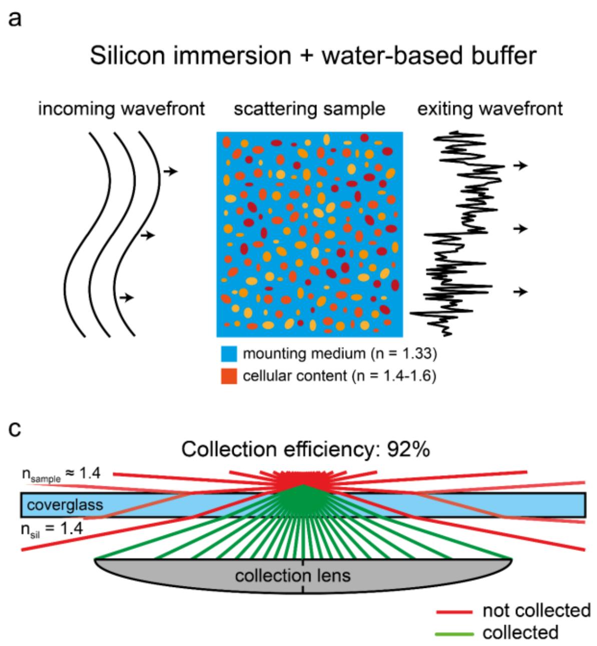

Illustration of the effect of a high refractive index buffer and objective choice. a) When using a water-based buffer the subcellular content of the tissue (organelles, DNA) has a large refractive index mismatch with the buffer. This rapid local change in refractive index drowns the gradually aberrated wave-front, thereby rendering AO less useful. b) By mounting the sample in a higher refractive index buffer using glycerol, the subcellular content causes less scattering and AO becomes more useful. c) A silicon immersion lens is the objective of choice when using a water buffer as the average refractive index of (brain) tissue is around 1.4. This matches the refractive index of the silicon oil, minimizing sample induced spherical aberration. However, the collection efficiency is only 92% as the largest available NA (1.35) is smaller than the refractive index. d) The glycerol-based buffer increases the average to around 1.48 (assuming a water content of 70%, which is replaced by the glycerol blinking buffer). Therefore a 1.49 NA oil immersion lens has a smaller refractive index mismatch than silicon oil and a higher collection efficiency as it collects the complete 2π sr solid angle and is therefore the objective of choice. For the computation of the collection efficiency we incorporated the Fresnel reflection at each interface.

Supplementary Figure 10

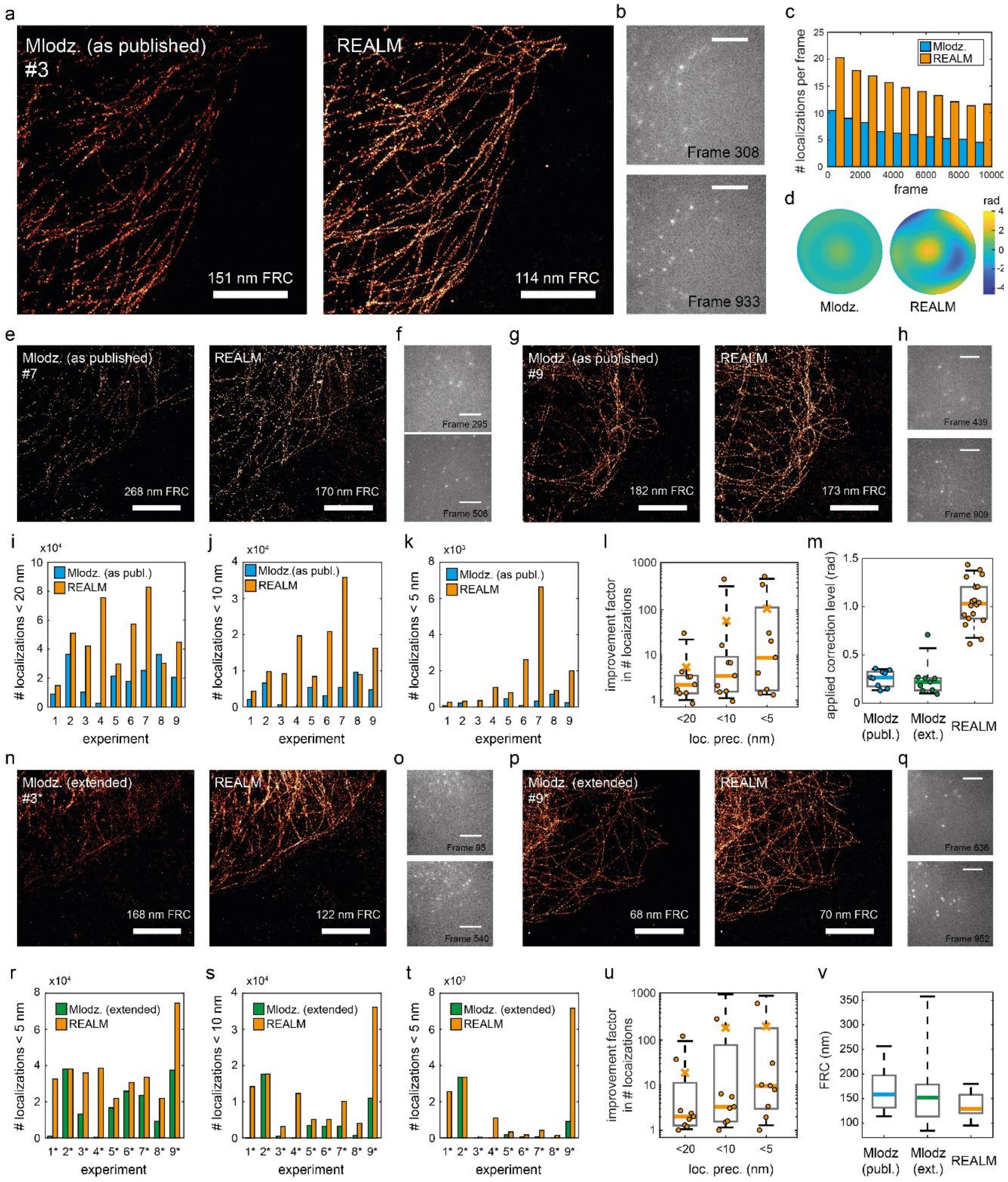

Direct comparison between the method proposed by Mlodzianoski et al. (here shortened to Mlodz.) and REALM by imaging COS-7 cells through 60 μm thick slices (repeated 2 × 9 times). We first performed both correction methods and performed SMLM while switching between mirror states during the acquisition every 500 frames. Mlodz. (as published) indicates the implementation as published, including a separate simplex routine with a different metric for primary and secondary spherical aberration, after which a secondary simplex routine corrects astigmatism and coma. Mlodz. (extended) denotes the method as published, except

that trefoil and secondary astigmatism are also included in the second simplex routine. REALM was implemented as in Supplementary Figure 9. a) Reconstruction of Mlodz. (as published) and REALM for experiment #3. b) Representative single acquisition for Mlodz. and REALM corresponding to the image shown in (a). Spots appear more confined and round with REALM. c) Average number of localization per frame for Mlodz. and REALM for (a). d) Estimated wave-front by Mlodz. (W_{rms} = 0.36 rad) and REALM (W_{rms} = 1.3 rad) for (a). e-h) Same as (a,b) but for experiment #7 & #9. i-k) Total number of successful localizations with a localization precision below 20 nm, 10 nm and 5 nm resp. for Mlodz. (as published) compared to REALM. l) Improvement factor in the number of localizations below 20 nm (2.2x, median), 10 nm (3.4x, median), and 5 nm (8.6x, median) with REALM compared to Mlodz. (as published). m) Applied correction level for Mlodz. (as published), Mlodz. (extended) and REALM. REALM applies a larger correction, indicating that simplex optimization remains in a noise induced local minimumn-u) Same as (e-l) but with Mlodz. (extended). v) Box plot of the calculated FRC of Mlodz. as published (151 nm median), Mlodz. Extended (145) and REALM (122 nm). The median FRC for all reconstruction with Mlodz. is 150 nm. All experiments were performed on three distinct samples. Ass scale bars indicate 5 μm. All box plots (l,m,u,v) indicate 9/91-percentile, 25/75-percentile and median. All reconstruction pairs are shown with the same contrast.

Supplementary Figure 11

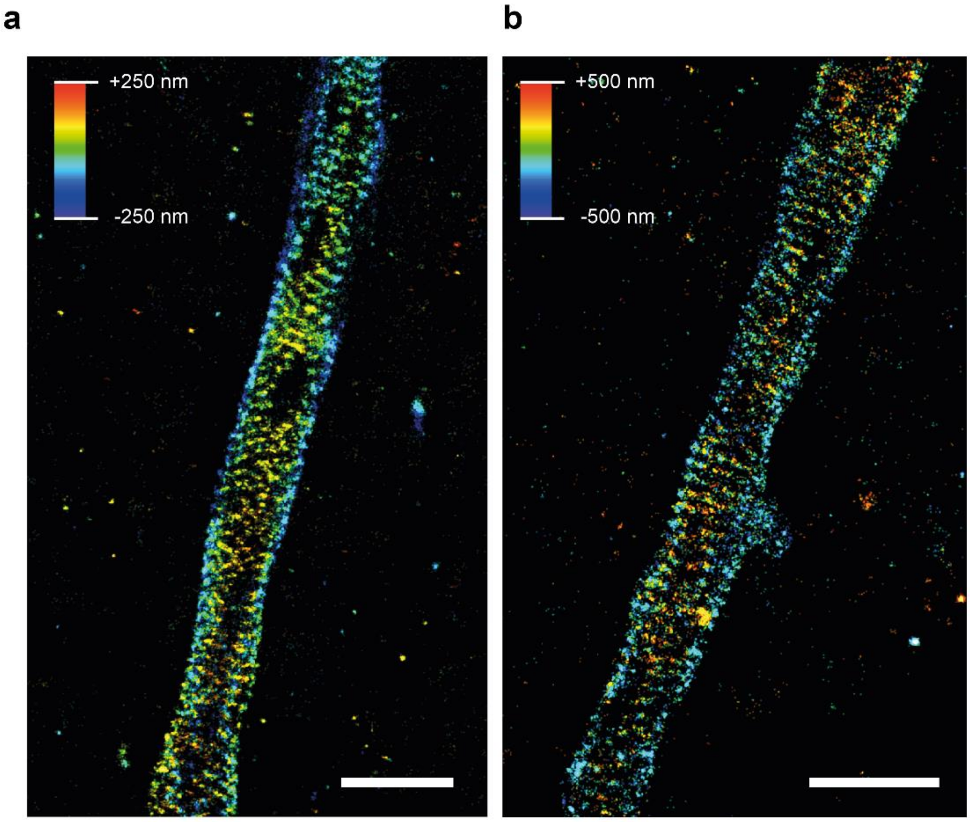

Example 3D SMLM reconstructions of βIV-spectrin in the AIS of layer 5 axons at a depth of 40 μm (a) and 50 μm (b). Scalebar indicates 2 μm. Repeated n=12 times in 4 distinct samples with similar results.

Supplementary Figure 12

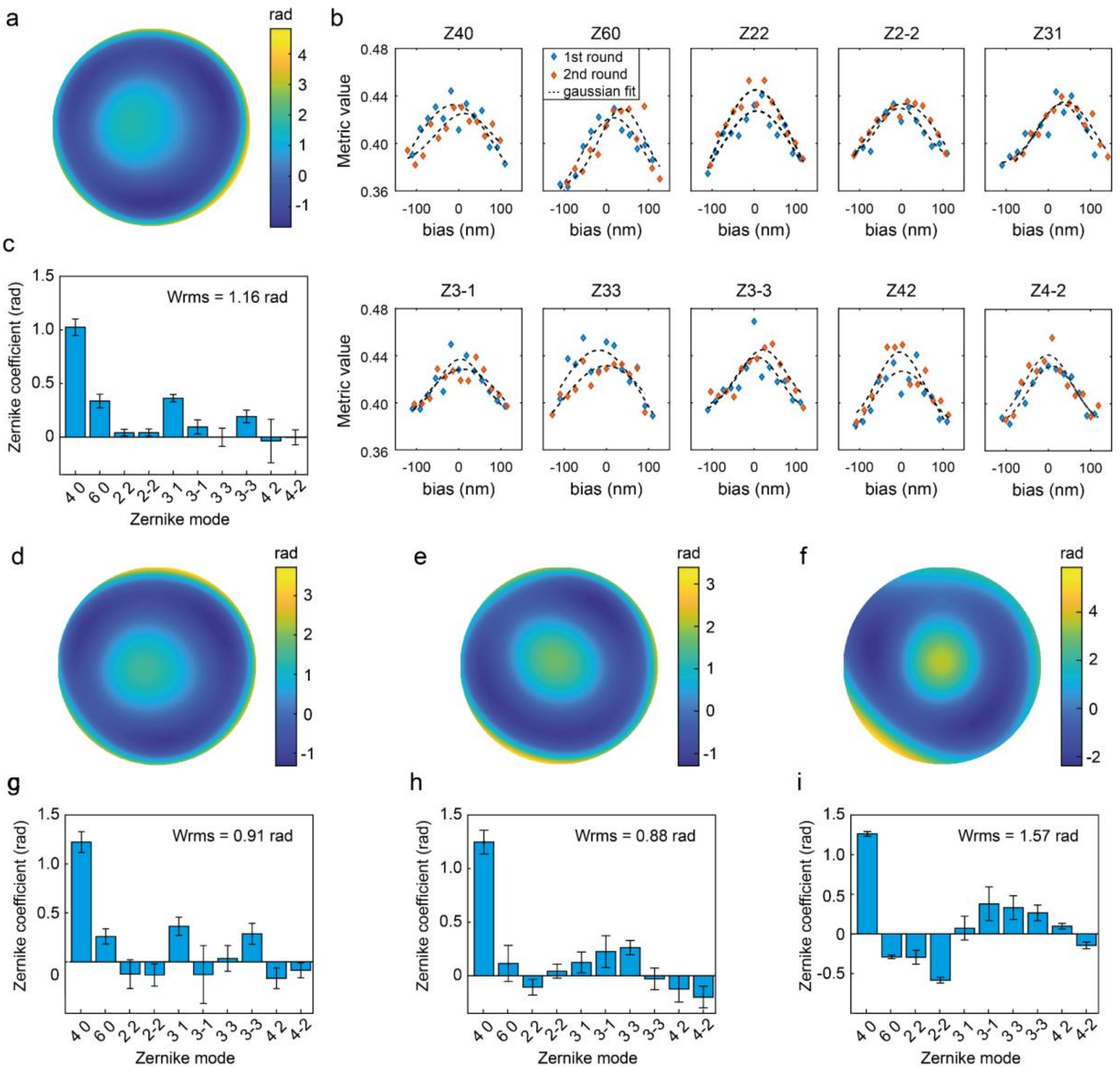

Estimated aberrations by REALM. a) Aberration profile corresponding to the βIV-spectrin reconstruction of Figure 3. b) Metric values and Gaussian fits of (a) of the model-based optimization algorithm of REALM. c) Estimated Zernike coefficients of (a) and (b). Error bars indicate the standard deviation based on the goodness of fit to the metric values. d-f) Aberration profiles corresponding to βIV-spectrin reconstructions of Supplementary Figure 11(a,b) and Figure 2 resp. g-i) Estimated Zernike coefficients of (d-f), respectively. Error bars indicate the standard deviation based on the goodness of fit to the metric values.

Supplementary Figure 13

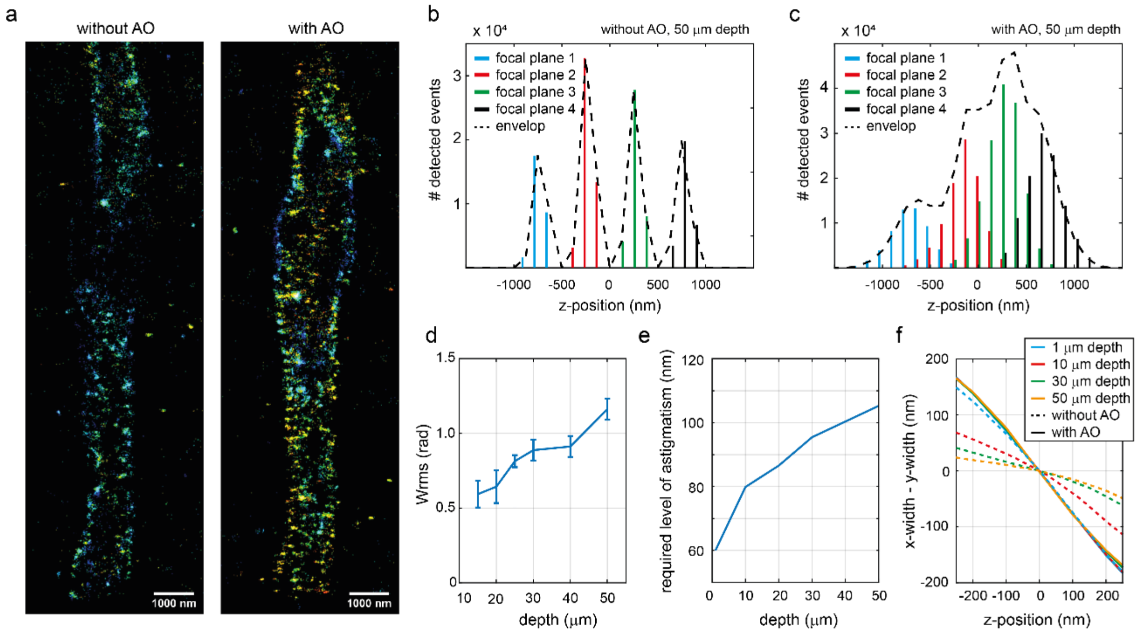

REALM improves 3D multiplane astigmatic SMLM. a) SMLM reconstruction of a single-plane astigmatic imaging experiment using βIV-spectrin at a depth of 50 μm. Every 500 frames the DM state was switched to system-corrected (without AO, 100 nm FRC) and sample-based correction (with AO, 76 nm FRC) (repeated n=3 times with similar results). b) Due to spherical aberration, the ellipticity of the PSF and therefore the z-encoding is lost without AO. This prevents multiplane astigmatic SMLM reconstructions to be 'stitched' together. c) Using REALM for aberration correction, z-encoding is restored allowing for 3D multiplane astigmatic SMLM. d) Measured aberration level using REALM on βIV-spectrin stained slices at different depths. Data comprised of estimated aberration levels in 3 samples. Error bars indicate standard deviation of REALM. Correction was performed once (n=1). e) Required level of astigmatism to maintain a similar calibration curve for astigmatic z-encoding, based on PSF simulations [19] and a refractive index of 1.48. f) Theoretical calibration curve with and without AO with astigmatism levels of (e) as a function of depth. Without AO the ellipticity of lost, resulting in failure of 3D multiplane astigmatic SMLM as shown in (b).

Supplementary Figure 14

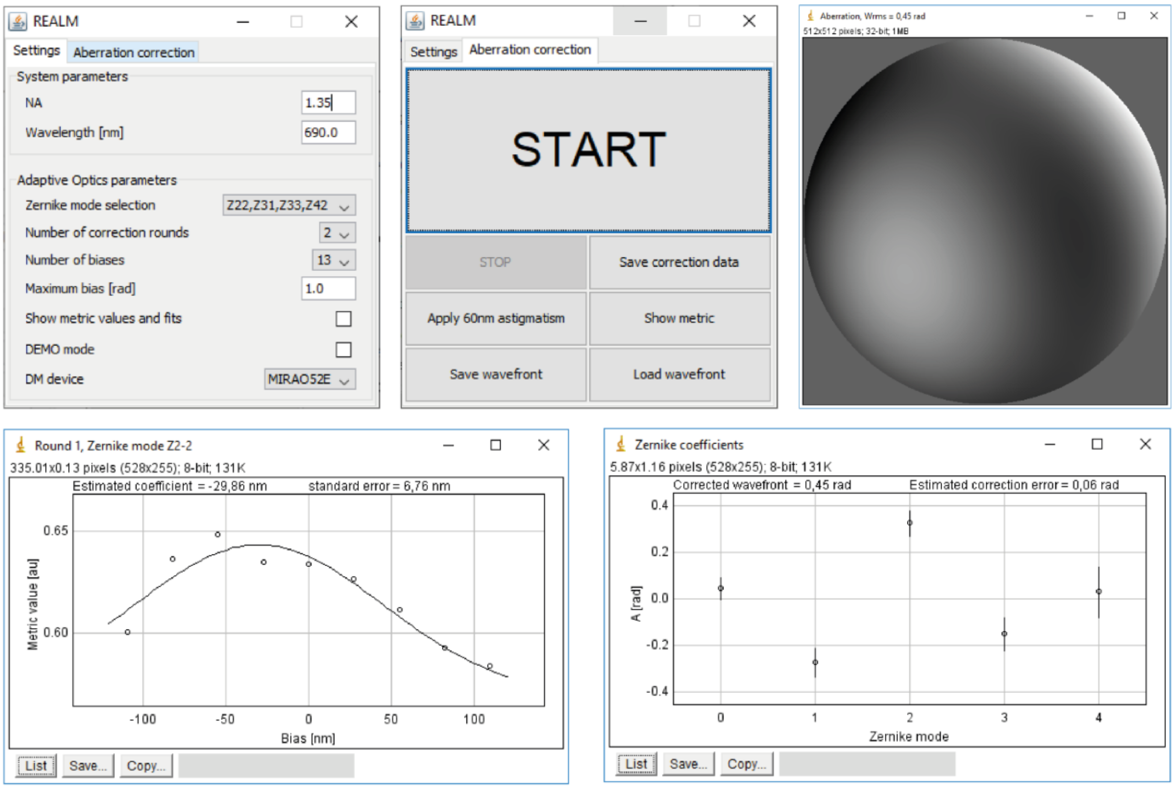

Images of the opensource Micro-Manager plugin REALM (https://github.com/MSiemons/REALM [28]). Relevant parameters can be tuned (Zernike modes, number of biases, maximum bias, number of correction rounds). REALM requires only little input parameters (NA and wavelength), resulting in a clear and user-friendly interface.
