# Supplementary material for: Robust adaptive optics for localization microscopy deep in complex tissue
Source: Nat Commun. 2021 Jun 7;12:3407. doi: 10.1038/s41467-021-23647-2 (PMC8184833; doi:10.1038/s41467-021-23647-2)
Supplement: Supplementary file 3 — Description of Additional Supplementary Files [file 41467_2021_23647_MOESM3_ESM.pdf]

Title: Supplementary Movie 1
Description: Improvement of REALM. SMLM acquisitions of Cos-7 cells immunostained for tubulin imaged through a 50 um thick brain section while switching between system corrected state (no AO) and sample-corrected state (with AO).

Title: Supplementary Movie 2
Description: Z-stack of 3D reconstruction of BIV-spectrin in a 350 um thick brain section at 50 um imaging depth.

Title: Supplementary Movie 3
Description: 3D render of 3D reconstruction of BIV-spectrin in a 350 um thick brain section at 50 um imaging depth.
