# Supplementary figures and images for: Robust adaptive optics for localization microscopy deep in complex tissue
Source: Nat Commun. 2021 Jun 7;12:3407. doi: 10.1038/s41467-021-23647-2 (PMC8184833; doi:10.1038/s41467-021-23647-2)

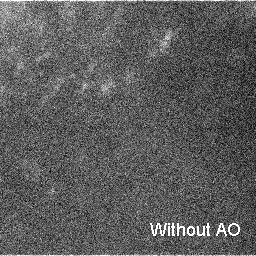

Supplement: Supplementary file 4 — Suppl. Movie 1 [file 41467_2021_23647_MOESM4_ESM.gif]

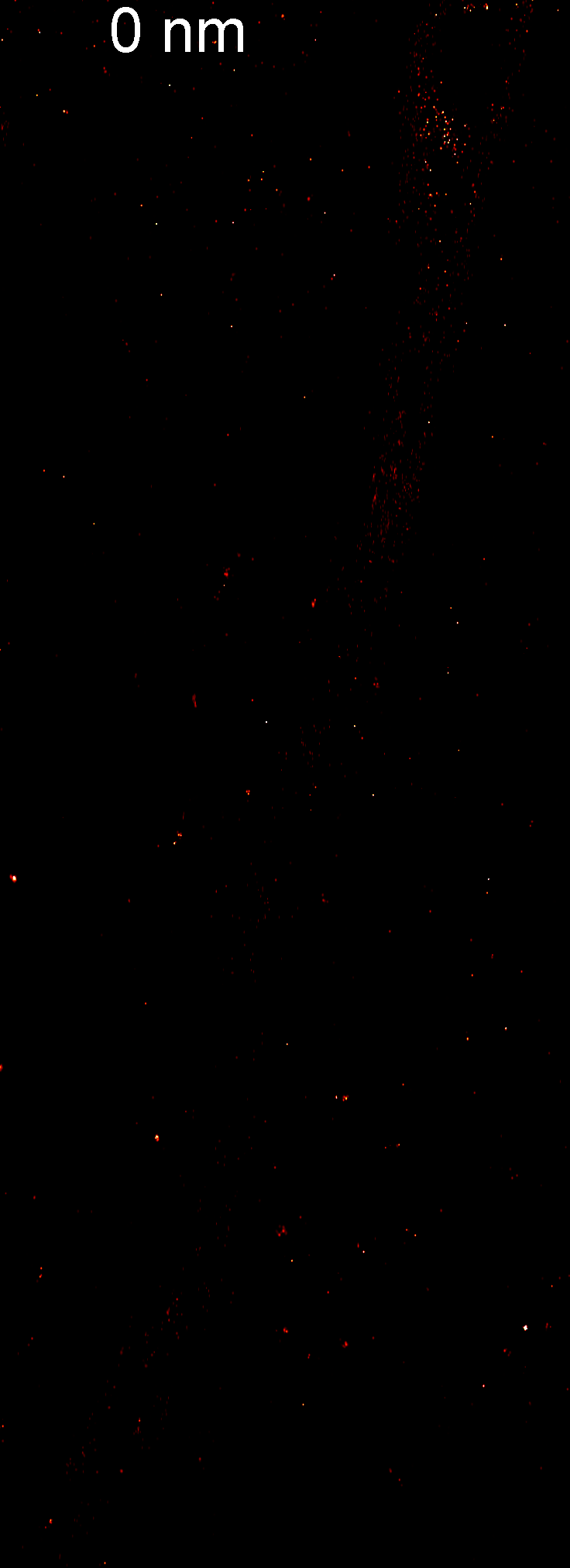

Supplement: Supplementary file 5 — Suppl. Movie 2 [file 41467_2021_23647_MOESM5_ESM.gif]

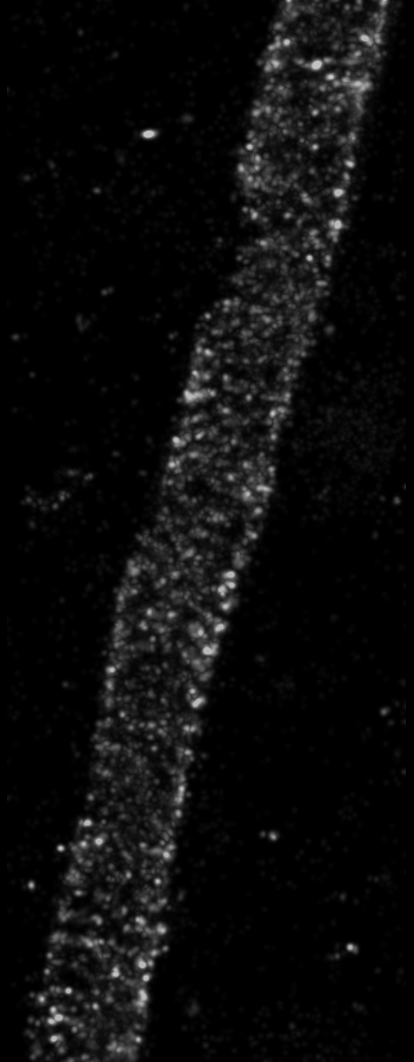

Supplement: Supplementary file 6 — Suppl. Movie 3 [file 41467_2021_23647_MOESM6_ESM.gif]
