# Supplementary material for: Robust adaptive optics for localization microscopy deep in complex tissue
Source: Nat Commun. 2021 Jun 7;12:3407. doi: 10.1038/s41467-021-23647-2 (PMC8184833; doi:10.1038/s41467-021-23647-2)
Supplement: Supplementary file 7 — Reporting Summary [file 41467_2021_23647_MOESM7_ESM.pdf]

nature research

Corresponding author(s): Lukas Kapitein

Last updated by author(s): Apr 9, 2021

Reporting Summary

Nature Research wishes to improve the reproducibility of the work that we publish. This form provides structure for consistency and transparency in reporting. For further information on Nature Research policies, see our Editorial Policies and the Editorial Policy Checklist.

Statistics

For all statistical analyses, confirm that the following items are present in the figure legend, table legend, main text, or Methods section.

n/a	Confirmed	
☐	☒	The exact sample size (*n*) for each experimental group/condition, given as a discrete number and unit of measurement
☐	☒	A statement on whether measurements were taken from distinct samples or whether the same sample was measured repeatedly
☒	☐	The statistical test(s) used AND whether they are one- or two-sided<br>*Only common tests should be described solely by name; describe more complex techniques in the Methods section.*
☒	☐	A description of all covariates tested
☐	☒	A description of any assumptions or corrections, such as tests of normality and adjustment for multiple comparisons
☒	☐	A full description of the statistical parameters including central tendency (e.g. means) or other basic estimates (e.g. regression coefficient) AND variation (e.g. standard deviation) or associated estimates of uncertainty (e.g. confidence intervals)
☒	☐	For null hypothesis testing, the test statistic (e.g. *F*, *t*, *r*) with confidence intervals, effect sizes, degrees of freedom and *P* value noted<br>*Give P values as exact values whenever suitable.*
☒	☐	For Bayesian analysis, information on the choice of priors and Markov chain Monte Carlo settings
☒	☐	For hierarchical and complex designs, identification of the appropriate level for tests and full reporting of outcomes
☒	☐	Estimates of effect sizes (e.g. Cohen's *d*, Pearson's *r*), indicating how they were calculated

Our web collection on statistics for biologists contains articles on many of the points above.

Software and code

Policy information about availability of computer code

Data collection	MATLAB 2017b was used for simulations (Fig 1) and MATLAB 2017b and Micro-Manager 1.4 with REALM plug-in (v1.0) for control of the set up.
Data analysis	Data analysis was performed with MATLAB 2017b with custom scripts and ImageJ using the DoM plugin for single-molecule detection and localization and the Fourier Ring Correlation plugin for computing the FRC resolution.

For manuscripts utilizing custom algorithms or software that are central to the research but not yet described in published literature, software must be made available to editors and reviewers. We strongly encourage code deposition in a community repository (e.g. GitHub). See the Nature Research guidelines for submitting code & software for further information.

Data

Policy information about availability of data

All manuscripts must include a data availability statement. This statement should provide the following information, where applicable:

- Accession codes, unique identifiers, or web links for publicly available datasets
- A list of figures that have associated raw data
- A description of any restrictions on data availability

The data that support the findings of this study are available from the corresponding author upon reasonable request.

Field-specific reporting

Please select the one below that is the best fit for your research. If you are not sure, read the appropriate sections before making your selection.

- [x] Life sciences
- [ ] Behavioural & social sciences
- [ ] Ecological, evolutionary & environmental sciences

For a reference copy of the document with all sections, see nature.com/documents/nr-reporting-summary-flat.pdf

Life sciences study design

All studies must disclose on these points even when the disclosure is negative.

Sample size	Sample sizes are indicated in the Figure captions.<br>For comparison between AO methods (Fig 1) , the sample size was maximized based on the stability of the DNA-PAINT sample and deformable mirror. Simulations indicated that beyond 25 samples the distributions did not change significantly.<br>For the comparison between REALM and Mlodzianoski et al. (Suppl. Fig 10) samples size was chosen such that the experiments could be performed in a reasonable amount of time.
Data exclusions	No raw data is excluded in this study
Replication	All findings of this study were replicated on different samples, leading to similar results: comparison between methods with DNA-PAINT: 2x, sandwich essays: 10x on 3 distinct samples(REALM vs no AO) and 18x on 3 distinct samples(REALM vs Mlodzianoski et al.), BIV spectrin stainings: 12x. Not all attempts at replication were successful, due manual errors such as incorrect focusing and staining issues.
Randomization	All comparisons in this study are performed and analyzed automatically with no user required input, randomization was therefore not required.
Blinding	All comparisons in this study are performed and analyzed automatically with no user required input, blinding was therefore not required.

Reporting for specific materials, systems and methods

We require information from authors about some types of materials, experimental systems and methods used in many studies. Here, indicate whether each material, system or method listed is relevant to your study. If you are not sure if a list item applies to your research, read the appropriate section before selecting a response.

Materials & experimental systems

n/a	Involved in the study	
[ ]	[x]	Antibodies
[ ]	[x]	Eukaryotic cell lines
[x]	[ ]	Palaeontology and archaeology
[ ]	[x]	Animals and other organisms
[x]	[ ]	Human research participants
[x]	[ ]	Clinical data
[x]	[ ]	Dual use research of concern

Methods

n/a	Involved in the study	
[x]	[ ]	ChIP-seq
[x]	[ ]	Flow cytometry
[x]	[ ]	MRI-based neuroimaging

Antibodies

Antibodies used	Mouse, anti alpha-Tubulin, monoclonal B-5-1-2, Sigma-Aldrich, T-5168-LOT1956565, Dilution 1:1000, was used for labeling the COS-7 cells.<br>Goat, anti-mouse igG(H+L), secondary antibody, Alexa647, Life Technologies, A21236-LOT038M4813V, Dilution 1:500, was used for labeling the COS-7 cells.<br>Rabbit, anti-BIV-spectrin, primary antibody, non-commercial, gift from M. Engelhardt, was used for labeling the brain sections.<br>Goat, anti-rabbit igG(H+L), secondary antibody, Alexa647, ThermoFisher, A21244-LOT2086730, Dilution (1:500 or 1:1000), was used for labeling the brain sections.
Validation	The anti alpha-Tubulin antibody (Sigma-Aldrich) was validated using Independent Antibody Verification (Demonstrating antibody specificity through the use of multiple antibodies against target in IHC or ICC.)<br>The bIV spectrin antibody produced by M. Engelhardt is affinity-purified and was validated first via Western blot (produced the published bands and no others, compared to Matt Rasband's original antibody which has the exact same sequence); then tested via numerous stainings co-labeled with other known AIS and noR markers, then also tested on tissue from bIV-spectrin null mice (no signal there).<br>This antibody has been subsequently used in several studies:<br>https://pubmed.ncbi.nlm.nih.gov/24653680/<br>https://pubmed.ncbi.nlm.nih.gov/29170630/

https://pubmed.ncbi.nlm.nih.gov/28922860/

Eukaryotic cell lines

Policy information about cell lines

Cell line source(s)	COS-7 cell line: Laboratory of Anna Akhmanova
Authentication	The COS-7 cell line is not authenticated.
Mycoplasma contamination	All cell lines used in this study tested negative for mycoplasma contamination (tested four times a year).
Commonly misidentified lines (See ICLAC register)	No commonly misidentified cell lines were used in this study.

Animals and other organisms

Policy information about studies involving animals; ARRIVE guidelines recommended for reporting animal research

Laboratory animals	In this study young-adult male Wistar rats (RjHan:WI) were used at an age between P21 and P35.
Wild animals	No wild animals were used in this study.
Field-collected samples	No field-collected samples were used in this study.
Ethics oversight	All animal experiments were performed in compliance with the European Communities Council Directive 2010/63/EU effective from 1 January 2013. They were evaluated and approved by the national CCD authority (license AVD8010020172426) and by the Royal Netherlands Academy of Arts and Science (KNAW) animal welfare and ethical guidelines and protocols (IvD NIN 17.21.01 and 19.21.11).

Note that full information on the approval of the study protocol must also be provided in the manuscript.
